# Comparing effectiveness of conservative policy to craniofacial surgery in children with metopic synostosis: protocol for an observational cohort study on clinical outcomes, psychosocial well-being and costs in a Dutch academic hospital

**DOI:** 10.1136/bmjopen-2024-094112

**Published:** 2025-05-06

**Authors:** Pauline Tio, Merel van Staalduinen, Jolanda Okkerse, K Dulfer, Nicole Erler, Sjoukje Loudon, Marieke Telleman, Tareq Abdel-Alim, Gennady Roshchupkin, Stella Heemskerk, Suzanne Polinder, Marie-Lise van Veelen, Natalja Bannink, Arianne van Driel, Mariët Faasse, Erwin Ista, K Joosten, Jochem Spoor, Elin Weissbach, Jacoba Kats, Sarah Lisa Versnel, Mieke Pleumeekers, Irene Mathijssen

**Affiliations:** 1 Department of Plastic and Reconstructive Surgery, Erasmus MC University Medical Center Rotterdam, Rotterdam, The Netherlands; 2 Department of Child and Adolescent Psychiatry/Psychology, Erasmus MC Sophia, Rotterdam, The Netherlands; 3 Department of Neonatal and Pediatric Intensive Care, Erasmus MC Sophia, Rotterdam, The Netherlands; 4 Department of Biostatistics, Erasmus MC University Medical Center Rotterdam, Rotterdam, The Netherlands; 5 Department of Epidemiology, Erasmus MC University Medical Center Rotterdam, Rotterdam, The Netherlands; 6 Department of Ophthalmology, Erasmus MC University Medical Center Rotterdam, Rotterdam, The Netherlands; 7 Department of Neurosurgery, Erasmus MC University Medical Center Rotterdam, Rotterdam, The Netherlands; 8 Department of Radiology and Nuclear Medicine, Erasmus MC University Medical Center Rotterdam, Rotterdam, The Netherlands; 9 Department of Public Health, Erasmus MC University Medical Center Rotterdam, Rotterdam, The Netherlands; 10 Child Brain Center, Erasmus MC Sophia, Rotterdam, The Netherlands; 11 Department of Pediatrics, Franciscus Gasthuis en Vlietland, Rotterdam, The Netherlands; 12 Department of Pediatrics, IJsselland Ziekenhuis, Capelle aan den IJssel, The Netherlands; 13 Dutch Patient and Parent Society for Craniofacial Conditions, Rotterdam, The Netherlands

**Keywords:** Cognition, Paediatric plastic & reconstructive surgery, Health Care Costs, Quality of Life, Neurosurgery

## Abstract

**Introduction:**

Traditionally, surgical intervention has been the standard treatment for children with metopic synostosis, assuming that it reduces the risk of raised intracranial pressure, thereby preventing vision and cognitive impairment, and also restores the abnormal head shape. However, recent research suggests a sporadic occurrence of raised intracranial pressure in patients with metopic synostosis. In addition, following surgery, an overall tendency to have worse cognitive and behavioral outcomes and more refractive errors compared to healthy peers is observed. Research on conservative (non-surgical) treatment in metopic synostosis is limited and lacks a comparative design. The purpose of this study is to compare the (cost-)effectiveness of conservative and surgical treatment in patients with metopic synostosis.

**Methods and analysis:**

This is the protocol for an observational cohort study with a duration of 8 years. A total of 450 patients with metopic synostosis will be included. The primary outcome is head growth as a predictor for increased intracranial pressure. Non-inferiority with regard to head growth from 0 to 8 years (yearly difference in SD) is determined using a linear mixed model adjusted for potential confounders. Secondary outcomes include papilledema, orthoptic outcomes; forehead shape; cognitive, behavioural and psychological outcomes; and societal costs. A cost-effectiveness analysis will be performed.

**Ethics and dissemination:**

The study has been reviewed and approved by the Medical Research Ethics Committee of the Erasmus MC, University Medical Center Rotterdam (MEC-2022-0142). Written informed consent will be obtained from both parents of each participant. The results will be disseminated by publication in international peer-reviewed journals.

**Trial registration number:**

ClinicalTrials.gov NCT06069479.

**STRENGTHS AND LIMITATIONS OF THIS STUDY**

- This is the first prospective cohort study evaluating different treatment policies in patients with metopic synostosis.
- This large cohort will provide information on clinical outcomes, psychosocial well-being and costs.
- This study will be conducted in a single academic centre.
- Randomisation of the type of treatment was not accepted by parents; therefore, an observational cohort study was chosen instead of a randomised trial.

## Introduction

 Premature closure of the metopic suture, also known as trigonocephaly or metopic synostosis, is the second most common type of craniosynostosis.^1 2^ The head shape in these patients is characterised by a wedge-shaped forehead, hypotelorism, temporal retrusion and biparietal widening.^2 3^ In contrast to other sutures of the calvaria, the fusion of the metopic suture early in life is a normal developmental process, with physiological closure occurring before 9 months of age.^4^,^6^

Traditionally, craniofacial surgery has been the standard treatment for these children, assuming that it reduces the risk of raised intracranial pressure, thereby preventing vision and cognitive impairment, and also restoring the abnormal head shape. There are two main options for surgical interventions, namely fronto-orbital advancement and endoscopic-strip craniectomy followed by helmet therapy. Recent research suggests that raised intracranial pressure occurs sporadically in these patients.^7 8^ Predictors for raised intracranial pressure include a decline in head growth and the presence of papilledema at funduscopy, which are described in 9% and 1.8% of surgically treated non-syndromic metopic synostosis patients, respectively.^7^ Although the second aim of surgery is to correct the abnormal head shape, a common long-term outcome observed after surgery is the recurrence of forehead deformities, occasionally resulting in a second surgical procedure.^8^,^10^

Patients with metopic synostosis have a higher risk of ophthalmologic, cognitive and behavioural problems. A higher prevalence of refractive errors is seen in patients with metopic synostosis compared with healthy controls.^11^,^13^ Patients with metopic synostosis experience hyperopia and astigmatism at rates of 22% and 23%, respectively, versus 8% and 4% in the age-matched norm population, which can contribute to headaches in these patients.^13^ Following surgery, patients with metopic synostosis score worse compared with healthy peers on several domains of cognitive and behavioural functioning.^14^,^19^

There are certain risks accompanying craniofacial surgery in these young patients. Complications occur sporadically, but they do occur, and include dural tears and wound infections.^8^,^10^ In addition, a blood transfusion is imperative in these patients when performing a fronto-orbital advancement. The coronal incision necessary with a fronto-orbital advancement results in a large scar for the child. Surgery is a stressful event not only for the child but also for the family and projects a significant amount of stress on the whole family.^20^ Caregivers of whom the child is planned for a surgical procedure can experience emotional distress and anxiety, which influences the child’s development.^21^,^24^

In recent years, the indication for craniofacial surgery in patients with metopic synostosis, particularly those with mild to moderate severity, has become a subject of debate.^25^ Conservative (non-surgical) management, involving regular follow-up appointments without surgical intervention, has gained interest due to the sporadic occurrence of signs indicating raised intracranial pressure. In a small group of patients that did not undergo surgery (n=40), none of these patients required surgical intervention for increased intracranial pressure during their follow-up.^8^ None of the existing literature has investigated the development of head shape over time in conservatively treated patients with metopic synostosis. It is hypothesised that a conservative policy allows for natural improvement of the abnormal head shape over time; however, the extent of the self-correction remains unknown.^26^ Conservative treatment could probably also remove the additional stress on the child and the family and the risk of complications associated with craniofacial surgery. While literature concerning cognitive outcomes in conservatively treated patients with metopic synostosis is limited and heterogeneous, there is a tendency for patients without surgical intervention to score slightly below average or exhibit a higher prevalence of concerns when compared with healthy controls.^14 17 27 28^ No research has been conducted in a large sample that directly compares cognitive and behavioural functioning between patients with metopic synostosis treated conservatively and those treated surgically.

Over the course of the past 6 years at Erasmus Sophia Children’s Hospital (Rotterdam, The Netherlands), approximately two-thirds (142/216) of parents of patients with metopic synostosis chose conservative treatment, while the remaining one-third opted for surgical treatment. None of the conservatively treated patients developed signs of increased intracranial pressure nor required craniofacial surgery. The choice of conservative treatment extends beyond its clinical consequences, influencing financial expenses associated with the management of patients with craniosynostosis. Although studies in the field of craniosynostosis have compared costs and established the cost-effectiveness of various surgical techniques,^29^,^31^ an evident gap exists in the literature concerning cost-effectiveness analyses comparing conservative and surgical treatments, particularly in patients with metopic synostosis.

Taking into account the sporadic occurrence of increased intracranial pressure and the overall tendency to have worse cognitive, behavioural and ophthalmologic outcomes even after surgery, the functional indication for surgical intervention for patients with metopic synostosis seems uncertain. In the existing literature, all outcomes following conservative treatment for patients with metopic synostosis are hard to determine due to small sample sizes, relatively short duration of follow-up and mild characteristics in the majority of the conservatively treated patients.^1426^,^28 32^ Therefore, a prospective cohort study with adequate follow-up is needed to determine if a conservative policy is as effective as surgical intervention. We present the study protocol for an observational cohort study on the effectiveness of a conservative policy compared with craniofacial surgery in metopic synostosis. This study presents a unique opportunity to assess differences in outcomes between conservatively and surgically treated patients with metopic synostosis in domains including intracranial pressure, vision, cognitive and behavioural functioning, impact on family and child, aesthetic outcomes and societal costs.

## Methods

### Patient and public involvement

The Dutch Patient and Parent Society for Craniofacial Conditions (LAPOSA) is a partner in the proposal and was involved in the design of the study. LAPOSA will also be involved in the dissemination of the results.

### Study design

Based on discussions with the patient society LAPOSA, an observational cohort design was chosen as the study design instead of a randomised trial, because randomisation of the type of treatment is not accepted by parents.

### Setting

In the Netherlands, treatment for craniosynostosis is fully reimbursed by the National Health Insurance programme. The care for patients with craniosynostosis is centralised in the Netherlands in two centres, with Erasmus Sophia Children’s Hospital treating over 80% of the Dutch population. This study is taking place at Erasmus Sophia Children’s Hospital. To evaluate the feasibility of transitioning a portion of follow-up care to non-specialised centres, follow-up appointments at the ages of 5 and 7 years are conducted in non-specialised hospitals (Franciscus Gasthuis and Vlietland, Rotterdam and Schiedam, The Netherlands, and IJsselland Ziekenhuis, Capelle aan den IJssel, The Netherlands).

### Eligibility criteria

Patients diagnosed with metopic synostosis at Erasmus Sophia Children’s Hospital will be recruited in the clinic. Eligible patients are up to 3 years of age and are diagnosed with either non-syndromic or syndromic metopic synostosis. These patients will be offered the opportunity to participate in the study by their clinician. Patients are excluded if they present with a metopic ridge only. Patient with multisuture craniosynostosis are excluded.

### Interventions

The study protocol aligns with our current clinical protocol up until the age of 8 years, except for additional questionnaires. At our centre, as of 2017, treatment decisions are made through a shared decision-making process in which parents can choose between two treatment options: conservative treatment or surgical treatment. Conservative treatment involves a nonsurgical approach with yearly routine follow-up appointments. The choice of the type of surgical treatment depends on the age at presentation and parental preferences, with two options available: fronto-orbital advancement and endoscopic-strip craniectomy with helmet therapy. If parents opt for a conservative policy, surgery is only performed if raised intracranial pressure occurs.

All patients with metopic synostosis receive identical follow-up care, irrespective of whether they undergo surgical or conservative treatment. This entails yearly hospital visits until the age of 8 years, followed by subsequent visits every 3 years until the age of 18 when craniofacial growth is considered to have reached its final stage. Head growth is measured every visit and funduscopy is performed annually up to the age of 4 years. Assessment of refractive errors occurs at 1, 4 and 8 years of age. Psychological screening is routinely offered between the ages of 2 to 8 years. 2D and 3D imaging is performed every other year at the ages of 0, 2, 4, 6 and 8 years.

### Outcomes

#### Clinical outcomes

Online supplemental A provides a visual overview of the clinical outcomes. When available, supplementary retrospective patient data will be collected in addition to prospective data.

##### Head circumference

The primary outcome is the change in head circumference, as head growth decline is an indicator for raised intracranial pressure. Head circumference is repeatedly measured every year from age 0–8 years. Measurements are performed manually with a measuring tape by skilled clinicians. Head circumference is defined in cm and corresponding SD based on national normative values. A decline in head circumference of more than 0.5 SD is considered clinically relevant. Head circumference is a significant predictor of intracranial volume, making it a very useful clinical measurement to monitor skull growth.^33 34^ As a non-invasive measurement accessible at every age, it serves as a valuable and efficient measurement to initiate further screening if abnormal in metopic synostosis patients.^7^ Patients with stagnation of the head circumference require further screening for raised ICP. Serial head circumference measurements, combined with a comprehensive screening protocol if necessary, provide a robust and clinically relevant approach to monitoring these patients. This method allows for regular, non-invasive assessment while minimising radiation exposure, which is particularly important in paediatric populations.

##### Papilledema

Funduscopies are performed annually by a paediatric ophthalmologist in children up to the age of 4 years to detect the presence (or absence) of papilledema, as an indicator for raised intracranial pressure.

##### Orthoptic outcomes

A full orthoptic examination is performed at the age of 1, 4 and 8 years by a paediatric orthoptist. The examination provides data on the refractive error (myopia, hyperopia, astigmatism), visual acuity, strabismus and amblyopia. Visual acuity scores are converted to logMAR; hyperopia, myopia and astigmatism are measured in diopters; presence of strabismus is measured in degrees; amblyopia is assessed as present or absent.

##### Forehead shape

Forehead shape is assessed at the ages of 0, 2, 4, 6 and 8 years using 2D and 3D photogrammetry and a visual analogue scale (VAS) score determined by the parents. Within the ERN CRANIO, a core outcome set for metopic synostosis has been developed, based on 2D photos.^35^ Serial 2D and 3D photos during follow-up will illustrate and quantify the growth pattern of the forehead over time. Comparison of the objective data (2D and 3D photos) with the subjective data (VAS score) will show how realistic parents experience their child’s forehead shape.

### Cognitive, behavioural and psychological instruments

Table 1 offers an overview of the cognitive, behavioural and psychological instruments. For a more detailed description of all psychometric properties, see online supplemental B. Questionnaires will be sent through email at pre-specified times and completed online using GemsTracker.

**Table 1:** Cognitive, behavioural and psychological instruments

Assessment/questionnaire	0 y	2 y	4 y	8 y
Development & cognition				
ASQ-extended	x			
BSID-III-NL		x*		
WPPSI-IV-NL			x*	
WISC-V-NL				x*
School performance (CITO)			x†	x†
Emotion, behaviour & psychosocial				
SDQ		x	x	x
Post-traumatic stress				
KJTS	x	x	x	x
PCL-5	x	x	x	x
Impact on family & child				
Interview	x	x	x	x
OBVL	x	x	x	x
CBSK				x‡
Decisional Conflict Scale	x			
Decisional Regret Scale				x
PedSQL		x	x	x§
EQ-5D-Y-5L	x	x	x	x

* Assessments by psychologist.

† School reports provided by parents.

‡ Child-reported questionnaire.

§ Both parent-reported and child-reported questionnaire.

ASQ, Ages and Stages Questionnaire; BSID, Bayley Scales of Infant and Toddler Development; CBSK, Competentiebelevingsschaal voor kinderen; CITO, Centraal Instituut voor Toetsontwikkeling; EQ-5D-5L-Y, EuroQol Five Dimensions Health Questionnaire Youth; KJTS, Kinder- en Jeugd Trauma Screener; OBVL, Opvoedingsbelasting vragenlijst; PCL-5, post-traumatic stress disorder checklist for DSM-5; PedsQL, Pediatric Quality of Life Inventory; SDQ, Strength and Difficulties Questionnaire; WISC, Wechsler Intelligence Scale for Children; WPPSI, Wechsler Preschool and Primary Scale of Intelligence.

#### Development & cognition

The cognitive and behavioural development of the children is evaluated at different ages using the following modalities:

At the age of 0 years old, the Ages and Stages Questionnaire-extended (ASQ-extended), a Dutch parent-reported computerised adaptive testing questionnaire for children aged 0–6 years which is adapted from the ASQ, is used to screen the child’s development.^36 37^ At the age of 2, 4 and 8 years, respectively, the Bayley Scales of Infant and Toddler Development (BSID-III-NL), Wechsler Preschool and Primary Scale of Intelligence (WPPSI-IV-NL) and Wechsler Intelligence Scale for Children (WISC-V-NL) are assessed by a psychologist. The BSID-III-NL is validated for children between the age of 2 weeks to 3.5 years and is widely used to assess neurodevelopment.^38^ The WPPSI-IV-NL is an intelligence test that is validated for children between the ages of 2 years and 6 months and 6 years and 11 months.^39^ The WISC-V-NL is an intelligence test that is validated for children between the ages of 6 years and 16 years and 11 months.^40^ For this study design, the ages of 4 and 8 years were selected for cognitive assessments due to their significance in the Dutch school system and the comprehensive set of outcomes measured at age 8. For further clarification on this decision, see online supplemental B. At the age of 4 and 8 years old, school performance is assessed with the nationwide Centraal Instituut voor Toetsontwikkeling (CITO) score to determine performance in elementary school.^41^ The CITO scores are provided by parents.

#### Emotional, behavioural and psychosocial functioning

The Strength and Difficulties Questionnaire (SDQ) is a brief behavioural screening questionnaire.^42^ The parent-reported version of the SDQ will be sent to parents when their child is 2, 4 and 8 years. The Self-perception Profile for Children (*Dutch*: Competentiebelevingsschaal voor kinderen (CBSK)) is a child-reported questionnaire validated for children between the age of 8 and 12 years, which is focused on how children perceive their own capabilities.^43^ The questionnaire is filled in by the child at the age of 8 years old.

#### Post-traumatic stress

The Kinder- en Jeugd Trauma Screener (KJTS) is used to screen for post-traumatic stress disorder (PTSD) in the child.^44^ KJTS is the Dutch validated version of the Child and Adolescent Trauma Screen (CATS). The parent-reported version is completed by the parents when their child is 0, 2, 4 and 8 years old. The Dutch PTSD Checklist for DSM-5 (PCL-5) is a self-reported questionnaire used to screen for post-traumatic stress disorder in adults.^45^ The PCL-5 is sent to the parents when their child is 0, 2, 4 and 8 years old.

#### Impact on family and child

Multiple questionnaires are used to measure the impact on the family and the child. The Parenting Stress Questionnaire (*Dutch*: Opvoedingsbelasting vragenlijst (OBVL)) is a questionnaire focused on child-parent relationship and parenting stress.^46^ The OBVL is sent to parents when their child is 0, 2, 4, and 8 years old. The Dutch Decisional Conflict Scale (DCS) measures parental perceptions of uncertainty in choosing options and effective decision-making.^47 48^ The DCS is sent to parents after the treatment decision with a window of 8 weeks. The Decisional Regret Scale (DRS) is distributed to parents when the child is 8 years old to measure distress or remorse after the treatment decision.^49 50^ The Pediatric Quality of Life Inventory (PedsQL) is a questionnaire measuring the health-related quality of life in children.^51^ The parent proxy-report form is sent to parents at the age of 2, 4 and 8 years old and the child proxy-report to the child at the age of 8 years old. The EuroQol Five Dimensions Health Questionnaire Youth (EQ-5D-Y-5L) measures the quality of life of children validated from 4 to 15 years.^52^ The parent-reported version is sent to parents when the child is 0, 2, 4 and 8 years old. Semistructured interviews with both parents separate are performed when their child is 0, 2, 4 and 8 years old, discussing the following aspects: parental concerns, parental stress indicators, traumatic experiences, hospital experience, relevant family factors, relation between parents and child, impact of disease on the child and family and decision-making process.

### Resource use and costs

All related societal costs will be taken into account, including costs related to healthcare resource use and loss of productivity for the parents for sick leave. This will allow for a comparison of the costs for both types of treatment. Healthcare resource use is extracted from the medical system, and in addition, the validated parent-reported iMTA Medical Consumption Questionnaire (iMCQ) will be used to measure healthcare consumption (ie, medical specialist care, hospitalisation and extramural healthcare consumption) and other costs directly associated with the treatment. Productivity losses are assessed by the iMTA Productivity Costs Questionnaire (iPCQ). Costs will be calculated by multiplication of healthcare consumption volumes by the cost prices per resource unit. Cost prices for healthcare resources use will be primarily derived from the Dutch manual on costing research.^53^ Cost prices of surgery will be determined by bottom-up micro-costing method. Productivity costs will be assessed using the friction cost method.^54^

### Power & sample size considerations

Due to the minimal extra time required from participants and parents, the inclusion rate is expected to be high and the loss to follow-up is expected to be low. Annually, around 50 new patients with metopic synostosis are referred to our centre, with an anticipated consent rate of 90% among parents, demonstrating their recognition of this observational study’s significance and their willingness to participate. In addition, within the first study year, children aged 1–3 years old will be included for follow-up with sufficient available retrospective data. Because at Erasmus Sophia Children’s Hospital, standard care for patients with metopic synostosis includes follow-up until the age of 18 years, drop-out rates are expected to be low. Inclusion will add up to 450 patients total.

A power calculation for the primary endpoint was performed using simulation. To obtain parameters for the simulation, a linear mixed model for age-adjusted SD scores of head circumference was fitted on existing data of children who underwent surgery. The model included a random intercept and (linear) slope for the child’s age at the time of the measurement to account for correlation between repeated measurements of the same child and to allow for child-specific trajectories. To take into account the non-linear shape of the children’s SD over time, a natural cubic spline with four df for age at the time of measurement was used in the fixed effects. The parameters from this model formed the assumption for the surgery arm in the power analysis simulation. For the conservative treatment arm, we assumed the SD at baseline follows the same distribution as in the surgery arm, but assumed linearly decreasing SD values over time. The rate of SD decrease in the conservative arm was increased over different simulation scenarios to find the most extreme scenario for which non-inferiority of the conservative arm could be shown with sufficient power.

Each simulated data set contained 245 and 195 children in the conservative and surgery arm, respectively. The number of available observations at each measurement time decreased with increasing age, taking into account the sequential inclusion of children throughout the study period (and resulting differences in length of follow-up). The differences in SD scores between subsequent measurements were calculated and modelled using a random-intercept linear mixed model that had the treatment arm as the only fixed effect. The resulting parameter estimate for the treatment arm describes the difference in the yearly decline of SD score in the conservative arm compared with the surgery arm. Non-inferiority was defined as the lower bound of the 95% CI of the treatment effect estimate being larger than −0.5 SD.

Assuming an average yearly decline in head circumference SD score of −0.25 in the conservative arm resulted in 90% power to demonstrate non-inferiority of the conservative arm at a 2.5% one-sided significance, with a non-inferiority margin of −0.5 yearly SD difference.

### Patient recruitment and timeline

Patients are informed by their clinician about the ongoing research and are offered the opportunity to participate in the study. Upon expressing interest, parents will be approached by an independent researcher who will provide them with detailed information about the study. Interested parents will be asked to sign the consent form indicating their willingness to participate with their child (Consent Form, see online supplemental C). For all parents who decline participations or withdraw from the study, their reasoning for making this decision will be documented. In order to promote participant retention, parents will receive 10 euro gift cards for every complete set of questionnaires.

Enrolment of participants and their parents has started in September 2022. The study follow-up period will extend until either participants reach the age of 8 years or until the end of the inclusion period (September 2030), whichever comes first. Currently, there are 90 participants included in this study (September 2024).

### Data collection & management

Data will be handled confidentially and anonymously. After receiving the signed consent form from the parents, every participant receives a unique study number that is used to link the data to the child. The coordinating researcher safeguards the key to the code.

All data from the questionnaires will be collected with GemsTracker, a software package for the distribution of digital questionnaires. Parents of patients receive emails at appropriate times with a secured link to GemsTracker’s website to answer questionnaires digitally. Both the emails as well as reminders, if questionnaires remain incomplete, are sent automatically with a maximum of two reminders. All data from clinical follow-up will be collected from the medical records. The coordinating researcher will regularly monitor whether all data are registered timely and properly. The combined data from both GemsTracker and the medical records are collected in Castor, a secured database. Daily back-ups are made automatically. Storage of personal data will be in line with the Dutch General Data Protection Regulation. Data access control will be in the hands of the principal investigator. Research data will be preserved for 10 years, according to national law. In the case of discontinuation of a participant, only data collected up until that point will be included.

### Statistical methods

#### Primary outcome

The primary outcome measurement of the head circumference is transformed to an age-specific and sex-specific SD score, according to national norms. The yearly decline in head growth is chosen as the primary outcome since this continuous measure has more power, allowing us to adjust for possible confounders. This would not be possible when using binary outcomes with low prevalence (eg, presence/absence of papilledema at funduscopy). Non-inferiority with regard to head growth from 0 to 8 years (yearly difference in SD score) is determined using a linear mixed model adjusted for potential confounders (including severity of phenotype, sex, syndrome and parental factors) and comparing the lower bound of the 95% CI of the treatment effect estimate (conservative vs surgery) to the non-inferiority margin of −0.5 SD.

#### Secondary outcomes

The presence or absence of papilledema on funduscopy is analysed with a repeated measures logistic regression to compare difference between the two groups. Prevalence of orthoptic anomalies is compared between the two groups and compared with the norm data, using the χ^2^ test. If the number of cases allows for estimating parameters, a logistic regression model is used; otherwise, the outcomes are stratified by treatment arms. Pearson’s correlation coefficients are calculated to determine a correlation between the VAS and the 2D photo grading and the VAS and the 3D photo grading per time point. For all validated instruments, norm values are available, including cut-off levels. Comparison will be made for the outcomes of the instruments between the two treatment groups and with the norm data. For some of the above-mentioned variables, different instruments are used at various time points to measure a single construct. In this case, the (ordinal) scores obtained from the instruments will be compared between the two groups at each time point using an independent-sample t-test. In case of repeated measures of a construct using the same instrument, we will use mixed-model analysis to compare the change of the given outcome over time between the two groups. In the case of multiple analyses that target the same research question, multiple testing correction will be applied. We will control the type I error rate using Bonferroni correction. As far as possible, missing data will be imputed and the number of patients used for analysis at each stage of the study shall be reported.

#### Economic evaluation

An economic evaluation will be conducted from a societal perspective in accordance with the Dutch guidelines for economic evaluations in healthcare, in which healthcare costs, patient and family costs, and costs outside of the healthcare sector (ie, productivity costs of the parents related to paid work absenteeism) will be considered.^54^ The time horizon is 8 years to include all relevant costs and effects. The primary outcome (ie, head circumference) will be used as the effect measure in the cost-effectiveness analysis. The incremental cost-effectiveness ratio of surgery versus conservative treatment will be expressed as costs per case of decline in head circumference >0.5 SD.

### Data monitoring

In accordance with Erasmus MC guidelines, the conduct of the study will be monitored. Monitoring will be done by an independent resident or PhD candidate of the Plastic and Reconstructive Surgery Department of the Erasmus MC. Monitoring is performed yearly and includes the following: inclusion and dropout rates, informed consent, protocol compliance and reporting of severe adverse events.

The intervention is not experimental but rather standard of care and is not expected to have a significant risk of potential harm to the patients; therefore, there will be no data monitoring committee.

All adverse events reported spontaneously by the parent of the participant or observed by the investigator or the staff will be recorded and followed. Interim analysis is done for head growth in 2025 to verify that the prevalence of raised intracranial pressure is within the expected range, and continuation of the study is justified.

## Ethics and dissemination

This study complies with the Declaration of Helsinki and is reviewed and approved by the MREC of the Erasmus MC, University Medical Center Rotterdam (MEC-2022-0142). This is a non-WMO study, which is an observational study in which no action or behavior is imposed on the participants in the study. All amendments will be notified to the MREC. This research adheres to the Code of Conduct for Health Research and Medical Treatment Contracts Act.

Written informed consent is obtained from the child’s parent/legal guardian by the coordinating researcher. This is done sufficient time after study information was shared, and after answering any questions of the parents to satisfaction. The informed consent form also indicates how participant data is stored, shared and used.

No provisions about ancillary and post-trial care are in place as the Dutch healthcare system ensures all participants get the care they need through health insurance. In accordance with Dutch law, Erasmus MC has a liability insurance and a human subject insurance which provides cover for damage to research subjects.

The results of this study will be published in international peer-reviewed journals and presented at international conferences. Parents and patients will be informed about any publication accompanied by a brief summary in Dutch. The published outcomes of this study will be implemented into clinical practice and the Dutch guideline for craniosynostosis will be updated accordingly.

## Supplementary material

10.1136/bmjopen-2024-094112 online supplemental file 1

10.1136/bmjopen-2024-094112 online supplemental file 2

10.1136/bmjopen-2024-094112 online supplemental file 3
