# Supplementary material for: Comparing effectiveness of conservative policy to craniofacial surgery in children with metopic synostosis: protocol for an observational cohort study on clinical outcomes, psychosocial well-being and costs in a Dutch academic hospital
Source: BMJ Open. 2025 May 6;15(5):e094112. doi: 10.1136/bmjopen-2024-094112 (PMC12056623; doi:10.1136/bmjopen-2024-094112)
Supplement: online supplemental file 1 [file bmjopen-15-5-s001.docx]

**Supplement A – Clinical outcomes**

| Clinical outcomes | 0 y | 1Y | 2 y | 3 Y | 4 y | 5 Y | 6 Y | 7Y | 8 y |
| --- | --- | --- | --- | --- | --- | --- | --- | --- | --- |
| Head circumference | X | X | X | X | X | X | X | X | X |
| Papilledema |  | X | X | X | X | * | * | * | * |
| Orthoptic outcomes |  | X |  |  | X |  |  |  | X |
| ForeHead shape | X |  | X |  | X |  | X |  | X |

*only if a decline in head circumference occurs or the child experiences headaches.
