# Supplementary material for: Comparing effectiveness of conservative policy to craniofacial surgery in children with metopic synostosis: protocol for an observational cohort study on clinical outcomes, psychosocial well-being and costs in a Dutch academic hospital
Source: BMJ Open. 2025 May 6;15(5):e094112. doi: 10.1136/bmjopen-2024-094112 (PMC12056623; doi:10.1136/bmjopen-2024-094112)
Supplement: online supplemental file 2 [file bmjopen-15-5-s002.docx]

**Supplement A – Psychometric characteristics instruments**

A series of assessments and parent or self-reported measures were used to monitor neurocognitive and behavioral development, stress and impact on family and child:

ASQ-extended – Parent-report

The Ages and Stages Questionnaire is a parent-reported validated questionnaire used to screen the child’s development (1). The Dutch adapted version of the ASQ is used, which is a computerized adaptive testing questionnaire for children aged 0-6 years, showing good reliability and validity (2). This questionnaire is used to screen the child’s development in 5 domains: Communication, Gross Motor skills, Fine Motor skills, Problem-solving, and Personal-social development. Items are answered by parents with ‘yes’, ‘sometimes, and ‘never’ and are clarified with images. Dutch norms and percentile scores are available. The development is determine based on cut-off values of percentile scores: percentile </= 3% slowest development, and percentile >/=90% is fastest development.

Bayley Scales of Infant and Toddler Development – Third edition (Dutch)

The Bayley Scales of Infant and Toddler Development (BSID-III-NL) is a widely used assessment for developmental functioning in children between the age of 2 weeks to 3.5 years (3). Normative data is present for all children aged 16 days to 42 months and 15 days and divided over 17 age-groups. The assessment is categorized into 5 domains: Cognition (91 items), Language (consists of the subscales Receptive language (49 items) and Expressive language (46 items)), Motor (consists of the subscales Fine motor (66 items) and Gross motor (72 items)), Social-Emotional, and Adaptive behavior. The assessment of the Social-Emotional and Adaptive behavior scales rely on the response of the caregiver, whereas the Cognition, Language and Motor scales are administered by a psychologist. The administration of a subscale starts at a specific starting item based on the age of the child. If the patient had failed to successfully complete the first three consecutive items, the administrator went back to a previous starting item until the infant completed the three consecutive items successfully of a starting point. Full credit is given for items prior to the starting item. The administration continued until the infant had a score of 0 on five items in a row. Index scores have a mean of 100 (*SD* = 15) and subscales have a mean of 10 (*SD* = 3).

Wechsler Preschool and Primary Scale of Intelligence – Fourth edition (Dutch)

The Wechsler Preschool and Primary Scale of Intelligence (WPPSI-IV-NL) is an intelligence test that is validated for children between the ages of 2 years and 6 months to 6 years and 11 months (4). The WPPSI-IV-NL consists of 15 subtests that generates a Full Scale Intelligence Quotient (IQ) and five primary indexes: Verbal Comprehension Index (VCI), Verbal Spatial Index (VSI), Fluid Reasoning Index (FRI), Working Memory Index (WMI), and Processing Speed Index (PSI). Normative data is present for children aged 2 years and 6 months to 3 years and 11 months and for children aged 4 years to 6 years and 11 months. The mean scaled score for the Full Scale IQ and the indexes is 100 (*SD* = 15) and for the subtests 10 (*SD* = 3). This study includes the first 10 subtests to obtain a score on all five domains and a Full Score IQ. Each domain will be assessed with two subtests.

Wechsler Intelligence Scale for Children – Fifth edition (Dutch)

The Wechsler Intelligence Scale for Children (WISC-V-NL) is an intelligence test that is validated for children between the ages of 6 years and 16 years and 11 months (5). The WISC-V-NL consists of 14 subtests that generates a Full Scale IQ and five primary indexes: Verbal Comprehension Index (VCI), Visual Spatial Index (VSI), Fluid Reasoning Index (FRI), Working Memory Index (WMI), and the Processing Speed Index (PSI). The mean scaled score for the Full Scale IQ and the indexes is 100 (SD = 15) and for the subtests 10 (SD = 3). This study includes the first 10 subtests to obtain a score on all five domains and a Full Score IQ. Each domain will be assessed with two subtests.

Note: For this study design, the ages of 4 and 8 years were selected for cognitive assessments due to their significance in the Dutch school system and the comprehensive set of outcomes measured at age 8. While we acknowledge the potential discrepancies between WPPSI and WISC scores, as highlighted by Salonen et al. (2023), our primary objective is to compare cognitive development between conservative and surgical groups rather than analyze longitudinal changes (6). Both the WPPSI and WISC demonstrate good reliability for this purpose. We opted against an additional assessment at age 6 to avoid potential ceiling effects, as the WPPSI-IV-NL's upper age limit of 6 years and 11 months could impact results for children tested closer to age 7. Our approach ensures consistent and reliable cognitive assessments at key developmental stages while aligning with the study's main comparative goals.

SDQ – Parent-report

The Strength and Difficulties Questionnaire (SDQ) is a brief behavioral screening questionnaire, with a parent-report and teacher-report version (7, 8). This questionnaire has been validated for children aged 2-4 years and 4-16 years and has a parent- or teacher-report and a self-report version. The items are categorized into five subscales, each comprising five items. These subscales produce scores for Emotional Symptoms, Conduct Problems, Hyperactivity/Inattention, Peer Relationship Problems, and Pro-Social Behaviors. Each item is assessed on a three-point scale: "Not True," "Somewhat True," and "Certainly True." The total difficulties score is derived by summarizing the four scales mentioned above, excluding Pro-Social Behavior. The parent-report version of the SDQ is sent to the parents when their child is 2, 4, and 8 years old. Cut-off scores for the total difficulties score at 2 years old is 12 or higher, at 4 years old is 15 or higher and at 8 years old is 14 or higher. Cut-off scores for the Emotional Problem scale at 2 years old is 3 or higher, at 4 years old is 4 or higher and at 8 years old is 5 or higher. For the Conduct problem scale, the cut-off score at 2 years old is 4 or higher, at 4 and 8 years old its 3 or higher.

CBSK – self-report

The Self-perception Profile for Children (Dutch: Competentiebelevingsschaal voor kinderen (CBSK)) is a child-reported questionnaire validated for children between the age of 8 and 12 years, which is focused on how children perceive their own capabilities (9). The CBSK contains 36 items, which are divided over six scales: School Performance, Social Acceptance, Athletic Competence, Physical Appearance, Behavioral Conduct, and Self-Worth. Scale scores are converted to percentile scores. Scores lower than the 15th percentile or above the 85th percentile indicate an extreme high score or low score of the child’s own capabilities. The reliability of each scale is moderate to high.

KJTS – Parent-report

The Kinder- en Jeugd Trauma Screener (KJTS) is used to screen for posttraumatic stress disorder (PTSD) in children (10). KJTS is the Dutch validated version of the Child and Adolescent Trauma Screen (CATS). This questionnaire has a self-report at the age of 7 years or older and two parent-report versions, between the age of 3-6 years and 7 years or older. The parent-report version is completed by the parents when their child is 0, 2, 4, and 8 years old. The KJTS is divided in 3 parts and consists of 41 items. The KJTS has 16 items measuring traumatic events, 20 items measuring DSM-5 PTSD symptoms, and 4 items measuring psychosocial functioning. Items are answered with ‘Yes’ and ‘No’ or with a four-point scale ‘Never’, ‘Once in a while’, ‘Half of the time’, and ‘Almost always’. Cut-off values for this screening tool are determined for the parent-report version 3-6 years as ‘Normal, not at risk’ (=/<11), ‘Increased trauma-related stress symptoms’ (11-14), and ‘Increased risk on PTSD’ (>/=15). Cut-off values for this screening tool are determined for the parent-report version 7 years or older as ‘Normal, not at risk’ (<15), ‘Possible trauma-related symptoms’ (15-20), ‘Increased trauma-related stress symptoms’ (>/= 21), and ‘Increased risk on PTSD’ (>/=25).

PCL-5- Parent-report

The Dutch PTSD Checklist for DSM-5 (PCL-5) is a self-reported questionnaire used to screen for posttraumatic stress disorder in adults, which contains 20 items regarding PTSD symptoms (11). Items are rated by a Likert scale from 0 (not at all) to 4 (extremely), which results in a total score between 0-80. The items can be divided into four subscales which match the four symptom clusters for PTSD within the DSM-5: Cluster B (re-experiencing), Cluster C (Avoidance), Cluster D (negative alterations in cognition and mood) and Cluster E (hyper-arousal). The Dutch translation has an excellent internal consistency and reliability, and a high criterion validity (12). A score of 31 or higher and at least 1 symptom in cluster B and C and at least 2 symptoms in cluster D and E indicate PTSD.

OBVL – parent-report

The Parenting Stress Questionnaire (*Dutch*: Opvoedingsbelasting vragenlijst (OBVL)) is a questionnaire focused on child-parent relationship and parenting stress (13). The OBVL contains 34 items which are answered on a Likert scale from 0 (not true) to 4 (very true). The total score involves five subscales, including: Parent-Child Relationship Problems, Parenting Problems, Depressive Mood (parent), Parental Role Restriction, and Physical Problems (parent). The OBVL has an overall good reliability and a Cronback’s alpha between 0.74 and 0.87. The total score is converted to aged-corrected T-scores. A T-score between 60-63 indicate mild problems and a T-score of 64 or higher indicates substantial problems.

Decisional Conflict Scale - Parent-report

The Decisional conflict scale (DCS) measures perceptions of uncertainty in choosing options and effective decision making (14, 15). The DCS contains 16 items which are rated from 0 (strongly agree) to 4 (strongly disagree). The Dutch version of the DCS is divided into three subscales with moderate to good reliability (14). The subscales include: uncertainty about choosing among alternatives, factors contributing to uncertainty, and perceived effectiveness of the decision. The DCS is sent to parents after the treatment decision with a window of 8 weeks.

Decision Regret Scale - Parent-report

The Decisional Regret Scale (DRS) measures distress or remorse after a treatment decision (16, 17). It contains 5 items which are scored on a Likert scale ranging from 1 (completely disagree) to 5 (completely agree). This scale has a good internal consistency with a Cronbach's alpha between 0.81 to 0.92.

PedsQL – Parent-report and self-report

The Pediatric Quality of Life Inventory (PedsQL) is a questionnaire measuring the health-related quality of life in children (18). This questionnaire contains 23 items divided over 4 subscales: Physical Functioning, Emotional Functioning, Social Functioning, and School Functioning. Three different summary scores can be calculated: Total Scale Score, Physical Health Summary Score, and Psychosocial Health Summary Score. Higher scores indicate a better health-related quality of life. Different versions are available based on the child’s age and the respondent (child self-report and parent proxy-report). Both the Dutch version of the child self-report as well as the parent proxy-report show good reliability (19, 20). The parent proxy-report form is sent to parents when their child is 2, 4, and 8 years old and the child proxy-report is sent to the child at the age of 8 years old.

Referral

The results of the assessments (BSID-III-NL, WPPSI-IV-NL, and WISC-V-NL) will be communicated to the parents via telephone within 3-4 weeks post-assessment. Additionally, a detailed report of the results will be recorded in the patient’s medical file. In cases where infants score below -2 standard deviations, the psychologist will consult with the parents regarding the need for referral. The nature of the referral will depend on the specific index or subscale exhibiting the low score and may include a referral to a physiotherapist or further evaluation by a psychologist. Simultaneously, the psychologist will review the outcomes of the questionnaires with the parents, and any indicated referrals will be facilitated accordingly. Furthermore, if the psychologist suspects a behavioral disorder based on the anamnesis or behavioral observations during the assessments, this will be discussed with the parents to determine if further assessment is required.

**Table 1. Overview of assessments and questionnaires**

| Assessment/questionnaire | 0 y | 2 y | 4 y | 8 y |
| --- | --- | --- | --- | --- |
| Development & cognition |  |  |  |  |
| ASQ-EXTENDED | x |  |  |  |
| BSID-III-NL |  | x^a^ |  |  |
| WPPSI-IV-NL |  |  | x^a^ |  |
| WISC-V-NL |  |  |  | x^a^ |
| School performance (cito) |  |  | x^b^ | x^b^ |
| Emotion, BEHAVIOR & psychosocial |  |  |  |  |
| SDQ |  | x | x | x |
| POSTTRAUMATIC sTRESS |  |  |  |  |
| KJTS | x | x | x | x |
| PCL-5 | x | x | x | x |
| IMPACT ON FAMILY & CHILD |  |  |  |  |
| Interview | x | x | x | x |
| OBVL | x | x | x | x |
| CBSK |  |  |  | x^c^ |
| Decisional conflict scale | x |  |  |  |
| Decisional regret scale |  |  |  | x |
| Pedsql |  | x | x | x^d^ |
| EQ-5D-Y-5L | x | x | x | x |
| Cost-effectiveness analysis |  |  |  |  |
| iMCQ | x | x | x | x |
| iPCQ | x | x | x | x |

^a^ assessments by psychologist; ^b^ school reports provided by parents; ^c^ child-reported questionnaire; ^d^ both parent- and child-reported questionnaire

ASQ: Ages and Stages Questionnaire; BSID: Bayley Scales of Infant and Toddler Development; WPPSI: Wechsler Preschool and Primary Scale of Intelligence; WISC: Wechsler Intelligence Scale for Children; SDQ: Strength and Difficulties Questionnaire; KJTS: Kinder- en Jeugd Trauma Screener; PCL-5: posttraumatic stress disorder checklist for DSM-5; OBVL: Opvoedingsbelasting vragenlijst; CBSK: Competentiebelevingsschaal voor kinderen; PedsQL: Pediatric Quality of Life Inventory; EQ-5D-5L-Y: EuroQol five dimensions health questionnaire youth; iMCQ: medical consumption questionnaire; iPCQ: productivity costs questionnaire

**References**

1. Squires J, Twombly E, Bricker D, Potter L. The ASQ-3 user's guide. 3rd ed. Baltimore: Paul H. Brookes Publishing Co.; 2009.

2. Van Baar AL, Verhoeven M, Hessen D, De Paauw-Telman L, Krijnen LJG. Monitoring development from 0-6 years: an online system for scientists and practitioners standardized for Dutch children. Submitted. 2025.

3. Van Baar AL, Steenis LJP, Verhoeven M, Hessen D. Bayley-III-NL. In: B.V PAaI, editor. Amsterdam2014.

4. Hurks P, Hendriksen J. WPPSI-IV-NL. Wechsler Preschool and Primary Scale of Intelligence – Fourth Edition. Technische handleiding. . Amsterdam: Pearson; 2020.

5. Hendriks M, Ruiter S, Schittekatte M, Bos A. WISC-V-NL. Amsterdam: Pearson; 2018.

6. Salonen J, Slama S, Haavisto A, Rosenqvist J. Comparison of WPPSI-IV and WISC-V cognitive profiles in 6-7-year-old Finland-Swedish children - findings from the FinSwed study. Child Neuropsychol. 2023;29(5):687-709.

7. Goodman R. The Strengths and Difficulties Questionnaire: a research note. J Child Psychol Psychiatry. 1997;38(5):581-6.

8. Maurice-Stam H, Haverman L, Splinter A, van Oers HA, Schepers SA, Grootenhuis MA. Dutch norms for the Strengths and Difficulties Questionnaire (SDQ) - parent form for children aged 2-18 years. Health Qual Life Outcomes. 2018;16(1):123.

9. Veerman JW, Straathof MAE, Treffers DA, Van den Bergh BRH, Ten Brink TL. Handleiding Competentiebelevingsschaal voor Kinderen (CBSK). Lisse: Swets & Zeitlinger; 1997.

10. Kooij L, Lindauer R. Kinder- en Jeugd Trauma Screener (KJTS-NL). Nijmegen: Praktikon; 2021.

11. Boeschoten MA, Bakker A, Jongedijk RA, Olff M. PTSD Checklist voor de DSM-5 (PCL-5). Diemen: Uitgave Stichting Centrum ’45, Arq Psychotrauma Expert Groep; 2014.

12. Van Praag DLG, Fardzadeh HE, Covic A, Maas AIR, von Steinbüchel N. Preliminary validation of the Dutch version of the Posttraumatic stress disorder checklist for DSM-5 (PCL-5) after traumatic brain injury in a civilian population. PLoS One. 2020;15(4):e0231857.

13. Vermulst A, Kroes G, De Meyer R, Nguyen L, Veerman JW. Opvoedingsbelasting Vragenlijst (OBVL). Nijmegen: Praktikon; 2015.

14. Koedoot N, Molenaar S, Oosterveld P, Bakker P, de Graeff A, Nooy M, et al. The decisional conflict scale: further validation in two samples of Dutch oncology patients. Patient Educ Couns. 2001;45(3):187-93.

15. O'Connor AM. Validation of a decisional conflict scale. Med Decis Making. 1995;15(1):25-30.

16. Dolan JG. A method for evaluating health care providers' decision making: the Provider Decision Process Assessment Instrument. Med Decis Making. 1999;19(1):38-41.

17. Brehaut JC, O'Connor AM, Wood TJ, Hack TF, Siminoff L, Gordon E, Feldman-Stewart D. Validation of a decision regret scale. Med Decis Making. 2003;23(4):281-92.

18. Varni JW, Seid M, Rode CA. The PedsQL: measurement model for the pediatric quality of life inventory. Med Care. 1999;37(2):126-39.

19. Schepers SA, van Oers HA, Maurice-Stam H, Huisman J, Verhaak CM, Grootenhuis MA, Haverman L. Health related quality of life in Dutch infants, toddlers, and young children. Health Qual Life Outcomes. 2017;15(1):81.

20. Engelen V, Haentjens MM, Detmar SB, Koopman HM, Grootenhuis MA. Health related quality of life of Dutch children: psychometric properties of the PedsQL in the Netherlands. BMC Pediatr. 2009;9:68.
