# Supplementary material for: Comparing effectiveness of conservative policy to craniofacial surgery in children with metopic synostosis: protocol for an observational cohort study on clinical outcomes, psychosocial well-being and costs in a Dutch academic hospital
Source: BMJ Open. 2025 May 6;15(5):e094112. doi: 10.1136/bmjopen-2024-094112 (PMC12056623; doi:10.1136/bmjopen-2024-094112)
Supplement: online supplemental file 3 [file bmjopen-15-5-s003.docx]

**Supplement B – Consent form parent/guardian**

**Children with trigonocephaly: is surgery necessary?**

I have been asked to give consent for my child’s participation in this medical-scientific study:

Participant name (child): …………………….. Date of birth: __ / __ / __

- I have read the information letter for the participant/parents/caregivers. I was also able to ask questions. My questions were answered sufficiently. I had enough time to decide whether I want my child to participate.
- I understand that participation is voluntary. I also understand that I can decide at any time to withdraw my child from the study. I do not need to provide a reason for this decision.
- I give permission for the researcher to inform my child's general practitioner/specialist(s) about their participation in this study.
- I give permission for the researchers to collect and use my child's data. The researchers will only use this data to answer the research question of this study.
- I understand that, for research monitoring purposes, certain individuals may have access to all of my child's data. These individuals are mentioned in the information letter. I give them permission to review my child's data for this purpose.
- Please check "yes" or "no" in the table below:

| I give permission for my child's data to be stored and used for other research conducted by Erasmus MC, as described in the information letter. | Yes ☐ | No☐ |
| --- | --- | --- |
| I give permission for my child's data to be stored and used for other research on metopic ridges by Erasmus MC and other European craniofacial centers, as described in the information letter. | Yes ☐ | No☐ |

- I agree that my child will participate in this study.

Name parent/guardian**: ………………………………

Signature: ………………………………… Date: __ / __ / __

Name parent/guardian **: …………………….

Signature: ……………………………………… Date: __ / __ / __

-----------------------------------------------------------------------------------------------------------------

I declare that I have fully informed the above-mentioned person(s) about this study.

If any new information arises during the study that may influence the parent's or guardian’s consent, I will inform them in a timely manner.

Researcher (or representative) name: …………………………

Signature: ……………………… Date: __ / __ / __

-----------------------------------------------------------------------------------------------------------------

<*if applicable*>

Additional information provided by:

Name: ………………………………………..

Role: ………………………………………

Signature: ………………………………. Date: __ / __ / __

-----------------------------------------------------------------------------------------------------------------

* Cross out what does not apply

** If the child is younger than 16 years old, the parent(s) with legal custody or guardian(s) must sign this form. In addition, children aged 12 to 15 years who are capable of making independent decisions must also sign their own consent form.

*The parent/guardian will receive a full information letter along with a signed copy of the consent form.*
