# Supplementary material for: Comparing effectiveness of conservative policy to craniofacial surgery in children with metopic synostosis: protocol for an observational cohort study on clinical outcomes, psychosocial well-being and costs in a Dutch academic hospital
Source: BMJ Open. 2025 May 6;15(5):e094112. doi: 10.1136/bmjopen-2024-094112 (PMC12056623; doi:10.1136/bmjopen-2024-094112)
Supplement: Supplementary file 4 [file bmjopen-2024-094112.reviewer_comments.pdf]

PEER REVIEW HISTORY

BMJ Open publishes all reviews undertaken for accepted manuscripts. Reviewers are asked to complete a checklist review form and are provided with free text boxes to elaborate on their assessment. These free text comments are reproduced below.

ARTICLE DETAILS

Title (Provisional)

Comparing Effectiveness of Conservative Policy to Craniofacial Surgery in Children with Metopic Synostosis: Protocol for an Observational Cohort Study on Clinical Outcomes, Psychosocial Wellbeing, and Costs in a Dutch Academic Hospital

Authors

Tio, Pauline; van Staalduinen, Merel; Okkerse, Jolanda; Dulfer, K.; Erler, Nicole; Loudon, Sjoukje; Telleman, Marieke; Abdel-Alim, Tareq; Roshchupkin, Gennady; Heemskerk, Stella; Polinder, Suzanne; van Veelen, Marie-Lise; Bannink, Natalja; van Driel, Arianne; Faasse, Mariët; Ista, Erwin; Joosten, K; Spoor, Jochem; Weissbach, Elin; Kats, Jacoba; Versnel, Sarah Lisa; Pleumeekers, Mieke; Mathijssen, Irene

---

VERSION 1 - REVIEW

**Reviewer**	**1**
**Name**	**Bezuhly, Michael**
**Affiliation**	**Dalhousie University Faculty of Medicine, Surgery**
**Date**	**25-Nov-2024**
**COI**	**None**

Many thanks for the opportunity to review this research protocol. Briefly, the authors describe their plans for an observational cohort study comparing conservative and surgical management of patients presenting to their centre with metopic craniosynostosis. The authors will submit patients to a battery of assessments, including head circumference measurement, fundoscopy, orthoptic, 2D and 3D photogrammetry, cognitive and behavioural testing, and cost-effectiveness analysis. The tools selected are appropriate and thorough. The sample size estimates and statistical plan are provided in appropriate detail. That said, I have a few comments/concerns, which, if addressed would improve this proposed study before it is initiated.

1. Please remove the “(Cost-)” from before the word “Effectiveness” in your title. It will make citing this protocol more difficult and is redundant as you include the word “Costs” at the end of the title.

2. The choice of occipitofrontal head circumference (OFHC) as a primary outcome, specifically as an indicator of increased intracranial pressure, is potentially problematic. The authors provide a reference to their earlier work examining post-surgical outcomes of trigonocephaly correction and found a significant association between OFHC stagnation and incidence of intracranial hypertension. Their group has observed similar associations in post-surgical syndromic (Spruijt et al., 2015) and sagittal craniosynostosis (van de Beeten et al., 2023) patients. The observation of stagnated OFHC in postoperative patients has been reported by groups in the US, Japan and Australia and is largely attributable to secondary synostoses resulting from surgery. I am afraid I do not support the a priori concept that OFHC in conservative patients is an indicator of increased ICP, particularly in metopic craniosynostosis patients. Several groups have observed frontal lobe hypoplasia in trigonocephalic patients, suggesting that this specific synostosis is a distinct entity from other non-syndromic synostoses, one where the brain truly dictates the skull. This may also explain behavioural issues that persist in these patients. While I think using OFHC is reasonable as an outcome, to claim it is likely to be an indicator of intracranial hypertension in non-operative metopic craniosynostosis patients does not follow from the authors' own work. Performing a back-of-the-envelope statistical analysis using the assumptions the authors have made, the use of serial OFHC is attractive as it significantly reduces the sample size required. That said, I would encourage the authors to consider a CT (or rapid hydrocephalus MRI scan if concerned about radiation) at baseline and again at, say, 4 years. The authors have included all other analyses, why not a CT to assess for radiographic signs of raised ICP?

3. Presumably, the surgical cohort will include both strip craniectomy/helmet and FOA patients. The authors should provide additional details/clarifications on how they will manage these two surgical subpopulations. Will they be treated as separate cohorts? Combined?

**Reviewer**	**2**
**Name**	**Horton, Jo**
**Affiliation**	**Birmingham Children's Hospital**
**Date**	**11-Jan-2025**
**COI**	**None**

An interesting future study that is well needed to address the gap around conversative treatment.

I did have a couple of queries that may be addressed in the manuscript or clarified via a response:

1. According to the website, the ASQ-4 has not yet been published and is not due to be published for a couple of years. How are the authors going to use measure this study?

2. Why are the authors not assessing cognitive development at 6 years of age with the WISC V? I have some concerns about assessing a child at 4 years of age with the WPPSI and then at age with the WISC. Scores are often lower using the WISC-V. Please see Salonen et al (2023) paper (Child Neuropsychology, 29 (5) 687-709) regarding the drop in scores when going from the WPPSI-IV to WISC-V. This will be needed to be considered and accounted for in the study and results if it is not possible to assess children at the age of 6.

3. How do the researchers classify severity of the suture fusion?

4. Does the centre use scans to confirm diagnosis?

5. Exclusion criterion: Will the authors being excluding multi-suture craniosynostosis even if it involves the metopic suture?

VERSION 1 - AUTHOR RESPONSE

Reviewer 1: Dr. Michael Bezuhly, Dalhousie University Faculty of Medicine

1. Please remove the "(Cost-)" from before the word "Effectiveness" in your title. It will make citing this protocol more difficult and is redundant as you include the word "Costs" at the end of the title.
Thank you for your suggestion, the title has been edited. See page 1 for adjustments.

2. The choice of occipitofrontal head circumference (OFHC) as a primary outcome, specifically as an indicator of increased intracranial pressure, is potentially problematic. The authors provide a reference to their earlier work examining post-surgical outcomes of trigonocephaly correction and found a significant association between OFHC stagnation and incidence of intracranial hypertension. Their group has observed similar associations in post-surgical syndromic (Spruijt et al., 2015) and sagittal craniosynostosis (van de Beeten et al., 2023) patients. The observation of stagnated OFHC in postoperative patients has been reported by groups in the US, Japan and Australia and is largely attributable to secondary synostoses resulting from surgery. I am afraid I do not support the a priori concept that OFHC in conservative patients is an indicator of increased ICP, particularly in metopic craniosynostosis patients. Several groups have observed frontal lobe hypoplasia in trigonocephalic patients, suggesting that this specific synostosis is a distinct entity from other non-syndromic synostoses, one where the brain truly dictates the skull. This may also explain behavioural issues that persist in these patients. While I think using OFHC is reasonable as an outcome, to claim it is likely to be an indicator of intracranial hypertension in non-operative metopic craniosynostosis patients does not follow from the authors' own work. Performing a back-of-the-envelope statistical analysis using the assumptions the authors have made, the use of serial OFHC is attractive as it significantly reduces the sample size required. That said, I would encourage the authors to consider a CT (or rapid hydrocephalus MRI scan if concerned about radiation) at baseline and again at, say, 4 years. The authors have included all other analyses, why not a CT to assess for radiographic signs of raised ICP?

We appreciate the reviewer's thoughtful comments regarding the use of occipitofrontal head circumference (OFHC) as a primary outcome in our study. We would like to address several points raised:

1. While we acknowledge that OFHC is indeed not a direct measure of ICP, it serves as a valuable screening tool for potential intracranial hypertension as it is closely related to intracranial volume. In non-operated craniosynostosis patients, a decrease in intracranial volume due to reduced skull growth can indeed occur as a result of the synostosis itself. Skull growth stagnation is a well-known phenomenon observed in late-referred patients and is not solely related to additional postoperative suture closures. Abnormal skull growth is, therefore, the most reliable measure that can be easily determined annually from a young age onwards.

Literature indicates a very low risk on raised ICP in surgically treated trigonocephaly patients, but only descriptive data is available on the risk of raised ICP in unoperated patients. It is therefore necessary to prove the safety of conservative management, which is the aim of this study. We appreciate the suggestion of a CT scan at 4 years, but this approach would miss the intervening years of screening, and its sensitivity is unclear. Moreover, at that age, we have the option to perform OCT, which has proven to have good sensitivity in combination with annual fundoscopy and is radiation-free.

2. Our screening approach involves not only OFHC measurements but also fundoscopy. We do not consider abnormal skull growth as a definitive indicator of increased ICP, but rather as the most relevant measure to initiate further screening if abnormal. This additional screening would involve MRI, and if necessary, ICP measurement according to our clinical protocol.

3. Although we do agree with the reviewer that metopic synostosis is a distinct entity from other non-syndromic synostoses, with a possible intrinsic brain anomaly, we respectfully disagree with the term "frontal lobe hypoplasia" in relation to trigonocephaly. Our research has demonstrated a different developmental pattern in white/gray matter development in these patients. The total white matter volume was smaller with an accelerated growth pattern, similar to white matter growth patterns in autism. Total brain volume and intracranial volume were normal. Also, DTI and ASL studies have not shown abnormalities. There is a clear relationship between genetic pathogenic findings in patients with trigonocephaly and patients with developmental delays, which, combined with the white matter differences, suggests a possible developmental disorder of the brain in children with trigonocephaly. It's important to note that skull surgery does not address this underlying issue.

4. The characterization of our statistical analysis as "back-of-the-envelope" is something we have to disagree with. Our proposal, including the statistical analysis which is performed by a trained biostatistician, has undergone peer review by ZonMW and was selected in a highly competitive process. The statistical analysis was also peer-reviewed after our grant application. A linear mixed model is fitted on the yearly OFHC because dichotomous outcomes did not result in sufficient power.

In conclusion, OFHC is not a direct measure of ICP, however it serves as a valuable and efficient measurement to initiate further screening if abnormal. We believe that serial OFHC measurements, combined with our comprehensive screening protocol, provide a robust and clinically relevant approach to monitoring these patients. This method allows for regular, non-invasive assessment while minimizing radiation exposure, which is particularly important in pediatric populations. This demonstrates our stepped approach, trying to prevent direct use of more invasive tools.

3. Presumably, the surgical cohort will include both strip craniectomy/helmet and FOA patients. The authors should provide additional details/clarifications on how they will manage these two surgical subpopulations. Will they be treated as separate cohorts? Combined?

The surgical cohort in our study will indeed include both strip craniectomy with helmet therapy and fronto-ortbital advancement patients. For our primary analysis, the surgical cohort will be treated as a combined group, comparing the overall outcomes between the surgical and non-surgical patients. We will investigate differences between different types of surgery if the number of children with a particular type of surgery is large enough to allow for a meaningful analysis and interpretation of the results.

Reviewer 2: Dr. Jo Horton, Birmingham Children's Hospital

1. According to the website, the ASQ-4 has not yet been published and is not due to be published for a couple of years. How are the authors going to use measure this study?

The ASQ-4 is available in the Netherlands and is used for research purposes. Although no publications on the ASQ-4 have been released yet, a research group at Utrecht University has developed the Dutch ASQ-4 and Dutch norms for its use. This has enable us, along with many other researchers in the Netherlands, to utilize the ASQ-4 in our studies. While we considered using the already published ASQ-3, we opted for the ASQ-4 due to the duration of our research (until 2030). We anticipated that by then, the ASQ-4 might have gained preference over the ASQ-3.

2. Why are the authors not assessing cognitive development at 6 years of age with the WISC V? I have some concerns about assessing a child at 4 years of age with the WPPSI and then at age with the WISC. Scores are often lower using the WISC-V. Please see Salonen et al (2023) paper (Child Neuropsychology, 29 (5) 687-709) regarding the drop in scores when going from the WPPSI-IV to WISC-V. This will be needed to be considered and accounted for in the study and results if it is not possible to assess children at the age of 6.

The age of 4 and 8 years old are important ages in the Dutch school system. This is part of the reason why these ages were chosen to assess cognitive development. Moreover, the age of 8-years old has the largest set of outcomes measured, which makes it also a relevant moment to assess cognitive development. In agreement with the reviewer, we know that there are discrepancies in outcomes between the WISC and WPPSI. However, it should be noted that our main aim is to compare the conservative and surgical group. To compare the cognitive development between these two groups, a reliable assessment tool is necessary. Both the WPPSI and the WISC show good reliability. Our main aim is not to analyze longitudinal changes in cognitive development over time. If this was the case, we do agree that a similar assessment tool would be more appropriate. Introducing an additional measurement at the age of 6 years in this specific research protocol would result in another issue. The WPPSI-IV-NL can be used up until the age of 6 years and 11 months. Not all our patients will visit the clinic at exactly 6 years old, appointments can also take place at 6 years and 6 months for example. If a well-performing child would have his/her appointment at a later stage, a ceiling effect will be reached. This is something we want to prevent. Taking all of the above together, we chose to do assessments with the WPPSI at age 4 and WISC at age 8.

3. How do the researchers classify severity of the suture fusion?

Severity is classified into mild, moderate, and severe. This classification is determined based on 3D photogrammetry images.

4. Does the centre use scans to confirm diagnosis?
All patients undergo 3D photogrammetry scans. In order to decrease the amount of radiation in young children, a CT scan is only performed in patients who opt for surgery.

5. Exclusion criterion: Will the authors being excluding multi-suture craniosynostosis even if it involves the metopic suture?
The prevalence of intracranial pressure in patients with multi-suture craniosynostosis is completely different compared to those of patients with a single-suture craniosynostosis of the metopic suture. These patients have different clinical characteristics are indeed excluded from this research. For clarity, the manuscript is revised and this is added as exclusion criterion, see page 5.
